# Alcohol in Tuberculosis

**Published:** 1910-04

**Authors:** 


### Alcohol in Tuberculosis.

The widespread use of alcohol in the treatment of tuberculosis makes the conclusions of Dr. T. D. Crothers, published in the *Journal of the A. M. A.* for February 19th, important as a warning to those who continue to have faith in its efficacy in this disease. His summary is as follows:

"1. The particular poisonous action of alcohol begins with its dehydrating properties, extracting the water from the cell and tissue so rapidly as to produce irritation, then narcotism. The ethers of alcohol, such as chloroform and similar substances, act specifically on the sensory centers, paralyzing them and cutting off all activity. These ethers vary in their action and specific poisonous effects through a wide range, but are all dehydrating and paralyzing in their action.

"2. Alcohol acts specifically on nerve cells and nerve centers, cutting off the sensations and disturbing the capacity to judge and reason, particularly of the surroundings and of the relations to them. One fact, standing out above all others, has startling significance, namely, the highest and last-formed faculties of the human brain, the consciousness of right and wrong, the ethical conceptions of duty and responsibility, are the first to become anesthetized and paralyzed from the effects of alcohol.

"The third toxic effect of alcohol is its diminution of vital power and capacity to resist disease and degeneration of any form. The defenses of the body are diminished, and the conservative reserve powers lowered.

"Finally, the toxic action of alcohol, whether given in small or large doses, is not a matter of theory or opinion."

